# Infection Control in Anaesthesia

**DOI:** 10.1111/j.1365-2044.2008.05657.x

**Published:** 2008-09

## 1.0.Summary

(1)A named consultant in each department of anaesthesia should liaise with Trust Infection Control Teams and Occupational Health Departments to ensure that relevant specialist standards are established and monitored in all areas of anaesthetic practice.

(2)Precautions against the transmission of infection between patient and anaesthetist or between patients should be a routine part of anaesthetic practice. In particular, anaesthetists must ensure that hand hygiene becomes an indispensable part of their clinical culture.

(3)Anaesthetists must comply with local theatre infection control policies including the safe use and disposal of sharps.

(4)Anaesthetic equipment is a potential vector for transmission of disease. Policies should be documented to ensure that nationally recommended decontamination practices are followed and audited for all reusable anaesthetic equipment.

(5)Single use equipment should be utilised where appropriate but a sterile supplies department (SSD) should process reusable items.

(6)An effective, new bacterial/viral breathing circuit filter should be used for every patient and a local policy developed for the re-use of breathing circuits in line with manufacturer’s instructions. The AAGBI recommends that anaesthetic departments should consider changing anaesthetic circuits on a daily basis in line with daily cleaning protocols.

(7)Appropriate infection control precautions should be established for each anaesthetic procedure, to include maximal barrier precautions for the insertion of central venous catheters, spinal and epidural procedures and any invasive procedures in high risk patients.

## 2.0.Introduction

The Association of Anaesthetists of Great Britain and Ireland (AAGBI) published guidelines in 2002 on problems relating to infection control in anaesthetic practice. In addition to AAGBI Officers and Council members, representation included the Royal College of Anaesthetists and the Medicines and Healthcare Products Regulatory Authority (MHRA). This is an updated version of that guidance document.

Healthcare organisations now have a legal responsibility to implement changes to reduce healthcare associated infections (HCAIs). The *Health Act 2006* provided the Healthcare Commission with statutory powers to enforce compliance with the Code of Practice for the Prevention and Control of Healthcare Associated Infection (The Code). The Code provides a framework for NHS Bodies to plan and implement structures and systems aimed at prevention of HCAIs. The Code sets out criteria that mandate NHS bodies, including Acute Trusts, and which ensure that patients are cared for in a clean environment. Anaesthetists should be in the forefront of ensuring that their patients are cared for in the safest possible environment. Further advice can be obtained from the Department of Health (DOH) website http://www.clean-safe-care.nhs.uk.

## 3.0.General principles

Healthcare providers have responsibilities under the *Health and Safety at Work Act 1974*; offences against the Act are covered by Criminal Law. The *Control of Substances Hazardous to Health (COSHH) Regulations 1999* have been considered part of the *Health and Safety at Work Act*; the Act ensures that Trusts are responsible for the health and safety of their employees and others (including visitors and patients), and the control and management of the risk of infection. As part of the *Health Act 2006*, the Department of Health (DOH) released the *Code of Practice for the Prevention and Control of Healthcare Associated Infections* [1].

Trust Chief Executives are accountable for ensuring that care delivered within each Trust meets relevant standards. Trusts should have Infection Control Committees and Infection Control Teams responsible for preparing policies and monitoring compliance with appropriate standards. A microbiologist should be designated to provide advice on microbiological aspects of decontamination and sterilisation. The AAGBI Working Party recommends that a named consultant in each Department of Anaesthesia should liaise with the Infection Control Team and the Occupational Health Department to ensure that relevant standards are established and monitored in all areas of anaesthetic practice.

### The environment

Hospital environmental hygiene encompasses a wide range of routine activities that are important in the prevention of HCAI. The hospital must be visibly clean and acceptable to patients, visitors and staff. Statutory requirements must be met in relation to safe disposal of clinical waste, laundry and linen. Bed occupancy should ensure enough time between patient admissions to ensure adequate cleaning and decontamination of the patient area.

The Code of Practice enshrines in law the duty of Trusts to maintain a clean and appropriate environment for patients including the fabric of the building and related structures. Operating theatres and associated areas should be designed and maintained to the standards defined in *Health Building Note 26, Facilities for Surgical Procedures* published in 2004. Microbiological commissioning and monitoring of operating theatre suites should adhere to national recommendations.

### Standard precautions

Anaesthetists will be involved in the care of patients who may harbour potentially pathogenic organisms, which may not be obvious or readily identifiable. Precautions aimed at preventing the transmission of organisms between patient and anaesthetist or between patients must be a routine part of anaesthetic practice.

The AAGBI recommends the use of Standard Precautions, which incorporate additional safeguards for specific procedures and patients, including single-use gloves, fluid resistant masks with a transparent face shield and gowns [2]. Precautions are recommended for all patients regardless of their diagnosis or presumed infectious status and must be implemented when there is a possibility of contact with:

1. Blood.
2. All other body fluids.
3. Non-intact skin.
4. Mucous membranes.

Preventative measures should be based on the likelihood of an infectious agent being present, the nature of the agent and the possibility of dispersion, e.g. splashing. A standard set of precautions should be established for every invasive procedure (see below) with additional risk assessment of each patient to determine extra and specific precautions that may be appropriate.

### Hand hygiene

#### Anaesthetists must ensure that good hand hygiene becomes an indispensable part of their clinical culture

Hand-mediated transmission is the major contributing factor to infection associated with healthcare [3]. Effective hand decontamination immediately before every episode of direct patient contact will result in a significant reduction in the transfer of potential pathogens and a decrease in the incidence of preventable HCAI [4]. Despite consistent advice, staff often neglect hand hygiene when caring for patients.

At the start of every session, and when visibly soiled or potentially contaminated, hands must be washed with liquid soap and water. When there is no soiling, the Hand Hygiene Liaison Group advocates that staff should use an antimicrobial hand rub between patients or activities [5] as this is effective and quicker. It is vital to ensure that the whole hand and fingers (particularly the tips), are exposed to the hand rub. Antimicrobial hand rub is not effective in preventing cross infection with *Clostridium difficile*.

Trusts must ensure that sinks, soap and antimicrobial hand rubs are conveniently placed to encourage regular use. Watches and jewellery (including dress rings and wrist adornments) must be removed at the beginning of each clinical session, before regular hand decontamination begins. Cuts and abrasions must be covered with waterproof dressings, which must be changed as appropriate. Staff with dermatitis, psoriasis or other skin conditions should consult with the Occupational Health Department for further advice.

The Code of Practice states that policies should be directly tied to the clinical governance framework and regularly reviewed under a system of audit, revision and update.

### Gloves

It is important to undertake a risk assessment regarding the safe use of gloves. Although they may offer some protection against inoculation with blood-borne viruses, incorrect use of gloves could actually spread infection between patients.

Sterile gloves must be worn for invasive procedures and contact with sterile sites. Non-sterile examination gloves must be worn for contact with mucous membranes, non-intact skin and all activities that carry a risk of exposure to blood, body fluids, secretions and excretions. All blood and body fluids, substances, secretions and excretions may be considered to be potentially infective regardless of the perceived risk of the source.

Gloves must be worn as single-use items. They should be put on immediately before an episode of patient contact and removed as soon as the activity is completed, and before contact with fomites, including curtains, pens, clinical notes, keyboards and telephones. Gloves should be changed between patients and between different procedures on the same patient. Gloves must be disposed of as clinical waste and hands should be washed or decontaminated following the removal of gloves. It has been demonstrated that 98% of anaesthetists’ contact with patients’ blood could be prevented by routine use of gloves [6].

Gloves of an acceptable quality must be available in all clinical areas. Latex-free gloves must be available for use for staff or patients who have an allergy or sensitivity to rubber gloves.

### Facemasks

The use of facemasks to decrease the incidence of postoperative wound infection has been questioned [7, 8]. However, masks with a face shield should be worn when there is a risk of blood, body fluids, secretions and excretions splashing into the face and eyes. Masks must also be worn by anaesthetists when carrying out a sterile procedure under full aseptic conditions (see later). If worn, masks should not be taken down to speak and should be changed if they become damp or contaminated. Masks must only be handled by the ties. Correctly fitting facemasks may also give some protection to the anaesthetist against inhaling infected droplets from the respiratory tracts of patients with infectious respiratory diseases.

The Working Party is aware that the wearing of masks remains an area of contention in many Trusts and conflicting evidence may be cited [9, 10]. In view of the continuing controversy about the wearing of masks, the Working Party suggests that local protocols should be agreed about their use in hospitals.

### Theatre caps

Theatre personnel in most UK operating theatres wear disposable headgear although there is little evidence for the effectiveness of this practice except for scrub staff in close proximity to the operating field [11]. However, theatre caps should be worn in laminar flow theatres during prosthetic implant operations, and it is the Working Party’s view that their general use should continue.

### Theatre suits and gowns

The skin of staff working in the operating theatre is a major source of bacteria that have the potential for being dispersed into the air. Clean theatre suits should be available for all staff in theatre. Full body, fluid-repellent gowns should be worn where there is a risk of extensive splashing of blood, body fluids, secretions and excretions. Sterile gowns should be worn when invasive procedures are undertaken. Disposable plastic aprons are often worn on wards in situations where there is a risk of physical soiling of clothing in order to prevent transmission of infection between patients.

Contaminated clothing should be changed and safely discarded into an appropriate receptacle at the earliest opportunity.

There is little evidence to show that wearing surgical attire outside the theatre and returning to the theatre without changing increases surgical wound infection rates. With the widespread move to admission on the day of surgery, the times when anaesthetists will have to leave theatre have increased and repeated changing will impact on theatre efficiency. Local policies must be developed and reflect the necessity for ‘theatre discipline’ and allay perceived concerns of patients and visitors. Local policies should be agreed between all theatre users and Trusts must ensure adequate provision of appropriate clothing. The DoH guidance on Uniforms and Workwear [12] also recognises that there is little evidence that work clothes pose a significant hazard in terms of increased infection rates.

### Shoes and overshoes

Special footwear should be worn in the operating department and cleaned if contaminated or after every use. Trusts should ensure that a system for cleaning theatre footwear is in place in each theatre suite. Plastic overshoes may increase bacterial contamination of floors [13] and, in addition, hands become contaminated when overshoes are put on or removed. Their use is not recommended.

### Movement within the theatre complex

To reduce airborne contamination, general traffic in and out of the operating theatre itself should be kept to a minimum. Doors should be kept closed to ensure the efficiency of the ventilation system.

Moving patients on their beds into the operating theatre may increase the bacterial count on floors, but it is claimed that this is of little significance if bed linen is changed before transfer [14]. All used linen must be handled safely to minimise the risk of contamination of the environment and staff. Used bed linen must be handled with care to reduce the release of small fomite particles into the air – bed linen should be ‘bagged’ by the bed or patient trolley. The use of separate trolleys from ward to transfer area and transfer area to table has not been shown to have benefit [15] although this practice continues in many operating areas.

If entering the operating theatre itself, visitors should change into theatre suits and wear designated footwear.

### Order of patients

There should be a written hospital policy requiring accurate printed theatre lists to be available prior to the scheduled date. ‘Dirty cases’, i.e. patients likely to disperse microbes of particular risk to other patients, should be identified before surgery and theatre staff should be notified. These patients should be scheduled last on an operating list to minimise risk. Where this is not possible, the Hospital Infection Society (HIS) advises that a plenum-ventilated operating theatre should require a minimum of 15 min before proceeding to the next case after a ‘dirty’ operation [16].

The most probable routes for transmission of infection between successive patients are airborne or on items and surfaces that have been in contact with the patient. Appropriate cleaning of the operating theatres between all patients should be undertaken. Whenever there is visible contamination with blood or other body materials, the area must be disinfected with sodium hypochlorite (according to local protocols) and then cleaned with detergent and water. Floors of the operating room should be disinfected at the end of each session.

### Safe use and disposal of sharps

Accidental injury has long been recognised as an occupational hazard in the healthcare setting. Accidental inoculation with infected blood, however small in amount, presents a significant risk to anaesthetists.

In the UK, 16% of occupational injuries occurring in hospitals are attributed to sharps injuries. These are predominantly caused by needles and are associated mainly with venepuncture, administration of intravenous drugs and recapping of needles. These should be preventable by adhering to national guidelines and agreed standards [17]:

- Sharps must not be transferred between personnel and handling should be kept to a minimum.
- Needles must not be bent or broken prior to use or disposal.
- Needles and syringes must not be disassembled by hand prior to disposal.
- Needles should not be recapped or resheathed.
- Used sharps must be discarded into an approved sharps container at the point of use.
- The sharps container should be sealed and disposed of safely by incineration when about two-thirds full or in use for more than four weeks, whichever is sooner. Sharps containers must comply with BS 7320:1990 –‘Specification for sharps’.
- Blunt aspirating needles should be used for drawing up drugs.
- Needle protection devices may reduce needlestick injuries but require further evaluation before widespread use can be advised.

### Preventing contamination of drugs

Drugs and fluids require safe handling by anaesthetists, who should follow local protocols for preparation and administration to prevent contamination.

Syringes and needles are sterile, single-use items and, after entry or connection to a patient’s vascular system or attachment to infusions, a syringe and needle should be considered contaminated and used only for that patient. A syringe must not be used for multiple patients even if the needle is changed. Before use, prepared syringes and needles should be stored in a clean container and syringes capped to avoid contamination. After use or at the end of the anaesthetic, all used syringes with needles should be discarded into an approved sharps container.

Care must be taken when drawing up drugs. Single use ampoules should be discarded after the required amount of drug is drawn up and not re-used for subsequent patients. Ampoules can be kept for identification purposes and discarded at the end of the list. Multiple-use ampoules are not recommended.

All infusions, administration sets or items in contact with the vascular system or other sterile body compartments are for single-patient use. An aseptic technique should be used when preparing infusions and breaks/taps in lines should be kept to a minimum. Injection ports should be maintained with a sterile technique, kept free of blood and covered with a cap when not in use. Connections and injection ports in intravenous lines should be kept to a minimum. Three-way taps should be avoided if practicable. Needle-free Luer injection devices should be used to cover exposed female Luer injection ports. These are easily cleaned before and after drug administration, do not harbour blood in crevices and also reduce the need for needles, resulting in a reduction of needlestick injuries.

## 4.0.Anaesthetic equipment and infection control

Items of anaesthetic equipment may become contaminated either by direct contact with patients, indirectly via splashing, by secretions or from handling by staff. Contamination is not always visible and all used pieces of equipment must be assumed to be contaminated and disposed of or, if reusable, undergo a process of decontamination. The Code of Practice has specific requirements for the decontamination of surgical equipment and other equipment used in patient care. There is a need to designate a person who is responsible for ensuring equipment cleanliness.

### Single-use equipment

Where appropriate, single-use disposable equipment will remove the difficulties of re-use and decontamination procedures. The use of such equipment is to be encouraged. However, there are problems of cost, storage and disposal of single-patient use devices, and for some equipment there is no feasible disposable alternative. The balance between single-use items and re-usable equipment will require local determination based on an assessment of patient safety, the available facilities and cost. Packaging should not be removed until the point of use for infection control, identification, traceability in the case of a manufacturer’s recall, and safety.

A multidisciplinary research team at the University of Nottingham has carried out a study investigating the use and re-use of single-use devices in English NHS operating theatres. The published paper clarified some of the issues around single-use devices such as single-use equipment that must be immediately discarded after use, e.g. suction catheters, and some that may be re-used in the same patient in the same episode, e.g. disposable laryngoscope blades [18].

### Decontamination

Decontamination is a combination of processes including cleaning, disinfection and/or sterilisation used to make a re-usable item safe to be handled by staff and safe for further use on patients. Effective decontamination of re-usable devices is essential in reducing the risk of infection. ‘Guidance on Decontamination’ prepared by the MHRA and DoH provides guidelines for the safe reprocessing of medical devices [19, 20]. It is recommended that each department identifies a designated consultant who, in conjunction with the appropriate bodies in their Trust, will develop specific guidelines for anaesthetic practice which satisfy national recommendations and that these practices are audited on a regular basis.

## Decontamination processes

**Cleaning**– removal of foreign material from an item. This usually involves washing with a detergent to remove contamination followed by rinsing and drying. All organic debris, e.g. blood, tissue or body fluids, must be removed before disinfection or sterilisation, as its presence will inhibit disinfectant or sterilant from contacting microbial cells. Cleaning before sterilisation is of the utmost importance in the effectiveness of decontamination procedures in reducing the risk of transmission of prions.

**Low Level Disinfection**–kills most vegetative bacteria (except TB and endospores), some fungi and some viruses using disinfectants such as sodium hypochlorite, 70% alcohol and chlorhexidine.

**High Level Disinfection**–kills vegetative bacteria (not all endospores), fungi and viruses. With sufficient contact time (often several hours), these high level disinfectants may produce sterilisation, e.g. the use of aldehydes, peracetic acid and chlorine dioxide.

**Sterilisation**–A process used to render an object free from viable micro-organisms, including all bacteria, spores, fungi and viruses, with techniques such as autoclaving (but see *prions* later).

### Risk assessment

The choice of equipment and/or the level of cleanliness/disinfection/sterility required of re-usable items may be assessed against the risk posed to patients of transmission of infection during any procedure in which the equipment is employed. It has been proposed by the MHRA Microbiology Advisory Committee that three levels should be considered:

1. High Risk–the device will penetrate skin or mucous membranes, enter the vascular system or a sterile space–these devices require sterilisation.
2. Intermediate Risk–the device will be in contact with intact mucous membranes or may become contaminated with readily transmissible organisms–these devices require high level disinfection or sterilisation.
3. Low Risk–the device contacts intact skin or does not contact patient directly–these devices require low level disinfection or cleaning.

## Infection control policy

### Anaesthetic face masks

Although normally in contact with intact skin, these items are frequently contaminated by secretions from patients and have been implicated in causing cross-infection [21]; local disinfection is not normally effective^.^ These items should be single-use items or should be sterilised between patients by an audited Sterile Supplies Department in accordance with the manufacturer’s instructions.

### Airways and tubes

Oral airways, nasal airways and tracheal tubes should be of single-use type since they readily become contaminated with transmissible organisms [22] and blood [23]. Ideally, supraglottic airways should be of the single-patient use type but the re-usable design is in common use and many anaesthetists perceive it as being less traumatic. Therefore, a supraglottic airway designed for repeated use should be sterilised (in an audited SSD) no more often than the manufacturer recommends. A supraglottic airway used for tonsillectomy or adenoidectomy should not be used again (see section on Prion Diseases). The AAGBI recommends single-use supraglottic airways.

### Catheter mounts–angle pieces

It is recommended that these items are single-patient use type.

### Anaesthetic breathing systems

The AAGBI has previously recommended that ‘an appropriate filter should be placed between the patient and the breathing circuit (a new filter for each patient)’. Although it appears that pleated hydrophobic filters have a better filtration performance than most electrostatic filters, the clinical relevance of this has yet to be established [24, 25]. The MHRA have also warned of the difficulty in assessing the performance of breathing filters in MDA/2004/013.

Until 2001 manufacturers of disposable anaesthetic breathing circuits supplied these as non-reusable items. In practice, most UK departments of anaesthesia used these circuits for more than one patient or for more than one operating session in conjunction with the use of a new filter for each patient. In 2006, the MHRA re-emphasised in Devices Bulletin DB 2006(04) the clinical and legal implications of re-use of devices labelled for single use and the AAGBI firmly supports their recommendations.

Therefore, departments may follow the manufacturer’s recommendations for use for up to 7 days. However, to ensure consistency in the infection control process, the Working Party recommends that circuits are disposed of when the anaesthetic machine and monitors are cleaned (see below). The AAGBI recommends that anaesthetic circuits are routinely changed on a daily basis. If visibly contaminated or used for highly infectious cases, e.g. tuberculosis, the circuits should be changed between patients and safely discarded. No attempt should be made to reprocess these items.

### Anaesthetic machines

Routine daily sterilisation or disinfection of internal components of the anaesthetic machine is not necessary if a bacterial/viral filter is used between patient and circuit. However, manufacturers’ cleaning and maintenance policies should be followed, and bellows, unidirectional valves and carbon dioxide absorbers should be cleaned and disinfected periodically. All the surfaces of anaesthetic machines and monitors should be cleaned on a daily basis with an appropriate disinfectant or immediately if visibly contaminated.

### Laryngoscopes

As with anaesthetic facemasks, laryngoscopes are known to become contaminated during use. Current practices for decontamination and disinfection between patients are frequently ineffective, leaving residual contamination that has been implicated as a source of cross-infection [26, 27]. Blades are also regularly contaminated with blood [28], indicating penetration of mucous membranes, which places these items into a high-risk category. Proper cleaning of laryngoscope blades is of great importance before decontamination/sterilisation, particularly of residue around light sources or articulated sections. New purchases should be of a design that is easy to clean. Although repeated autoclaving may affect the function of laryngoscopes [29], the Working Party recommends that re-usable laryngoscope blades should be sterilised by an audited SSD between patients, following the manufacturers’ instructions. Plastic sheaths may be used to cover blades and handles to reduce contamination but it has been noted, especially with blade covers, that these have created difficulties during tracheal intubation.

There are an increasing number of inexpensive, single-use laryngoscope blades and handles of improving design available, and their use is to be encouraged. The choice of blade must be dictated by Departments of Anaesthesia. Traditional blades should be available at all times in case difficulty is encountered.

Laryngoscope handles also become contaminated with micro-organisms and blood during use, and they should be washed/disinfected and, if suitable, sterilised by SSDs after every use. The knurled handles of laryngoscopes cannot be cleaned reliably manually if covered in blood or body fluids.

Anaesthetists should show great care when handling laryngoscopes: wear gloves during intubation and place used instruments in a designated receptacle to prevent contamination of surfaces, pillows and drapes.

### Fibreoptic bronchoscopes

These are expensive items which cannot be autoclaved. Decontamination is dependent on sufficient contact time with high level disinfectants and it is particularly important that the washing and cleaning process removes all tissue residues from the lumens. Decontamination is best achieved with an automated system. National guidelines for care of these instruments have been provided by the MDA, now the MHRA, DoH and NHS Estates [18, 30, 31, and 32].

With the uncertainty of the future implications of variant Creutzfeld Jakob Disease (CJD), these items should have a unique identifier which should be recorded at every use to permit future tracing.

### Bougies

Re-use of these items has been associated with cross-infection [33]. Manufacturers recommend that a gum elastic bougie may be disinfected up to five times between patients and stored in a sealed packet. It is preferable that alternative single-use intubation aids are employed when possible.

### Surfaces

The surfaces of anaesthetic machines and monitoring equipment, especially those areas which are likely to have been touched by the gloved hand that has been in contact with blood or secretions, should be regarded as contaminated and should be cleaned at the earliest opportunity, probably between patients. Local policies should be in place to ensure that all equipment that touches intact skin, or does not ordinarily touch the patient at all, is cleaned with a detergent at the end of the day or whenever visibly contaminated. This includes non-invasive blood pressure cuffs and tubing, pulse oximeter probes and cables, stethoscopes, electrocardiographic cables, blood warmers etc., and the exterior of anaesthetic machines and monitors. Items such as temperature probes should be for single patient use.

### Oxygen masks and tubing

Single-patient use products should be used.

### Resuscitation equipment

Single-patient use equipment should be kept in a sealed package or should be resterilised between patients according to the manufacturer’s instructions. All training equipment should be handled similarly.

## 5.0.Prion diseases

Transmissible Spongiform Encephalopathies (TSEs) are a group of illnesses in which there is progressive neurological degeneration associated with characteristic pathological changes. TSEs are caused by abnormal prions, which are infectious proteins. Microscopic traces of tissue often remain on surgical instruments after washing and autoclaving or disinfecting and any prion protein in these traces could still transmit the disease if inoculated into another patient. However, successive washing after use reduces the concentration so that, after about 10 decontamination cycles, infectivity becomes negligible [34].

Variant Creutzfeldt-Jakob disease (vCJD) is a TSE caused by the same prion protein that causes Bovine Spongiform Encephalopathy (BSE) in cattle. After an incubation period of several years, the disease shows itself as a progressive degeneration of the brain leading to death. It is similar to sporadic CJD, which has been spread by neurosurgical instruments, dura mater grafts and pituitary extracts. However, while sporadic CJD prion protein is to be found only in brain, spinal cord and posterior eye, the prion protein of vCJD can also be found in lymph nodes, appendix and tonsils. Prion protein becomes detectable in tissues in the later period of incubation and is present in higher concentrations once the disease becomes manifest [34]. Advice on the detection and prevention of prion diseases is available from the Advisory Committee on Dangerous Pathogens (ACDP), the Spongiform Encephalopathy Advisory Committee (SEAC) and the National Institute for Health and Clinical Excellence [35]. The CJD Incidents Panel Advice advises on the prevention of transmission of CJD in specific cases [36, 37].

### Tonsillectomy and adenoidectomy

Lymphoid tissue, including that of the adenoid and tonsil, is of ‘medium infectivity’ for vCJD and it is possible that a patient undergoing surgery could be incubating this disease. Surgeons routinely operate with traceable reusable instruments in accord with latest national advice, since single-use instruments were associated with excessive bleeding and risks of CJD transfer are low [38]. Tracheal tubes, supraglottic airways and oral airways should be destroyed after use for these operations as they can be contaminated by infectious tissue and batch cleaning may cause transfer of protein from one device to another [39].

Theoretically, a laryngoscope blade used at the end of surgery or for emergency re-intubation could become contaminated. However, the Working Party’s estimate is that the risk is extremely small and probably less than those posed by the use of a disposable laryngoscope for what could be a difficult laryngoscopy. Therefore, the recommendation is that single-use laryngoscopes are not mandatory in anaesthesia for tonsil and adenoid surgery. Those purchasing laryngoscopes with single-use blades (and other single-use devices) have a responsibility to ensure that the performance of the instruments is at least as good as that of the standard reusable alternatives.

### Anaesthetic management of cases of CJD

This advice covers known cases, suspected cases and those who are at risk of developing CJD. Isolation is not needed. Standard (‘universal’) precautions are essential. Blood and other samples should be labelled ‘Biohazard’. Invasive procedures such as central venous cannulation and spinal anaesthesia mandate the use of full aseptic procedures, including mask and eye protection. The area around where invasive procedures are performed should be clear enough to allow easy mopping of spillages. High infectivity tissues are those in the brain, spinal cord and posterior eye; medium risk tissues for CJD are those in the anterior eye and olfactory epithelium–any needles or probes used by anaesthetists near these tissues will be disposable. In vCJD, lymphoid tissue is also classed as having medium infectivity. In these circumstances, it is advisable that any laryngoscope used after the tonsil has been operated on or any bronchoscope used for biopsy on patients in this group should be treated as potentially contaminated. These instruments should either be destroyed (easily done with single-use laryngoscopes) or should be quarantined pending advice. Such advice can be obtained from the CJD Incidents Panel.

### Transmission of vCJD by transfusion of blood and blood products

Although only three cases have been discovered, this is the only route of transfer of vCJD as yet shown to be from medical interventions. The National Blood Transfusion Service repeatedly updates its procedures so as to reduce the tiny risk of transfer of prions, viruses and bacteria [40]. Procedures include leucodepletion and sourcing most plasma products from outside the UK. Anaesthetists can further decrease the risk by minimising the use of blood and blood products.

### Summary: how to reduce the risk of transmission of prion disease

1. Adherence to the same universal precautions as for other potential infections.
2. Minimising the use of transfusion of blood and blood products.
3. Use of SSD decontamination and sterilisation with tracing for all reusable equipment.
4. Ensuring that any instrument that contacts the brain, spinal cord or dura is destroyed afterwards.
5. The employment of single-use equipment when this is as reliable and safe as the re-usable alternatives.
6. Destroying or quarantining instruments used on cases who may have CJD and for those known to be at risk.
7. Ensuring that all airway devices used during tonsillectomy and adenoidectomy are discarded after use.

## 6.0.Infection control precautions for anaesthetic procedures

Carrying out procedures in an operating theatre does not pose a lower risk of infection than other hospital locations and the risk of infection depends on the procedure and on the level of barrier protection rather than the surrounding environment [41].

### Maximal barrier precautions

Maximal barrier precautions involve full hand washing, the wearing of sterile gloves and gown, a cap, mask and the use of a large sterile drape [42]. The skin entry site should be cleaned with an alcoholic chlorhexidine gluconate solution or alcoholic povidone-iodine solution [43]. The antiseptic should be allowed to dry before proceeding.

Certain invasive anaesthetic procedures require this optimum aseptic technique:

- Insertion of central venous catheters.
- Spinal, epidural and caudal procedures.

The Working Party is aware that many anaesthetists do not employ this level of asepsis for ‘one-shot’ spinals or epidurals but believes that, when central neural spaces are penetrated, full aseptic precautions are required.

Comprehensive guidelines have been prepared for insertion and maintenance of central venous catheters [44] and are commended to all anaesthetists. The use of care bundles has been advocated by IHI (Institute of Health Improvement) to reduce catheter related bacteraemias. Originally developed in the USA, Care Bundles is an approach that systematically appraises clinical processes. It is based on measuring the actual provision of therapeutic interventions according to standards, informed by evidence, which local clinicians set themselves [45].

### Other barrier precautions

Certain invasive procedures do not require full barrier precautions as above but nevertheless demand appropriate aseptic techniques. Such precautions involve the wearing of sterile gloves and use of small drapes, although similar attention is required to hand washing and skin preparation. These procedures include:

- Peripheral regional blocks.
- Arterial line insertion.

Peripheral venepuncture or intramuscular injection in low-risk patients will involve handwashing, non-sterile gloves and skin preparation with propyl alcohol. Peripheral intravenous catheters are a significant source of nosocomial bacteraemias and care is required.

### High-risk patients

Certain patients may be especially vulnerable to infection, e.g. the immunocompromised, or offer particularly high risk of transmitting infection, e.g. tuberculosis and HIV. For the immunocompromised, maximal barrier precautions are required for all invasive procedures and similarly, where there is a high infection risk, staff should concentrate not only on preventing cross-infection between patients but in protecting themselves by ensuring compliance with all precautions.
